# The importance of early and accurate service information for young people accessing gender care

**DOI:** 10.1038/s41598-025-18532-7

**Published:** 2025-10-09

**Authors:** Meaghan Storey, Lynn Gillam, Fae Garland, Ken Pang, Simona Giordano

**Affiliations:** 1 Centre for Social Ethics and Policy, University of Manchester, Manchester, UK; 2 Department of Paediatrics, University of Melbourne, Melbourne, Australia; 3 Children’s Bioethics Centre, Royal Children’s Hospital, Melbourne, Australia; 4 Clinical Sciences, Murdoch Children’s Research Institute, Melbourne, Australia; 5 Department of Adolescent Medicine, Royal Children’s Hospital, Melbourne, Australia

**Keywords:** Health services, Medical ethics, Paediatrics

## Abstract

Relevant and accurate information is crucial for patient access to healthcare. In the current literature, the availability and provision of information have been proposed to be key barriers to young people accessing gender care. However, it is unclear what kind of information young people want and how clinical services should address this access barrier. Here, we share the results of qualitative interviews with 14 young adults (aged 18–22) who accessed gender care as minors (under 18 years old) in Australia. Three themes were identified from the data: (1) information seeking, (2) a lack of information affects expectations, and (3) a lack of information impacts decision making about accessing care. Participants looked for at least three types of information prior to accessing gender care: medical, experiential, and service information. Our results highlight the key gap was information about services and how they work. Clinical services should consider their practical and ethical role in providing accurate and reliable service information to young people, prior to the clinical encounter.

## Introduction

Young people and their families encounter a number of barriers when trying to access gender care. Gender care encompasses the various social, legal, and medical services that young people and their families may engage with when a young person experiences gender dysphoria^1^. One barrier to accessing gender care that has been identified in the literature is the availability and provision of information^2–4^. For example, young people are often unsure where to find information about clinical services^2^ and encounter clinicians who lack knowledge about care options or referral pathways^3,4^. Such patient experiences have been described as instances of “information marginalisation”^5^ and “informational erasure”^6^, respectively. Both terms aim to highlight the role institutions have played in the lack of information available to patients and the lack of clinician training and knowledge about particular patient groups. While the current literature has established a need for further training of clinicians around paediatric and adolescent gender care^3^, further research is needed to understand the impact limited or unclear information has on young people prior to the clinical encounter. Moreover, it is unclear what kind of information young people want at the early stages of seeking care and how clinical services might go about addressing this access barrier.

This paper investigates what young people know about the process of accessing paediatric gender care in Australia. Using findings from interviews with young adults (age 18–22) who accessed gender care as minors (under 18 years old) in Australia, we explore in particular what young people knew prior to accessing gender care and how the information they encountered during this time helped to shape their clinical experience. While our focus is on the Australian context, information provision and its availability as a barrier to accessing care has been reported internationally^3,4^. Our findings are therefore broadly relevant to clinicians and clinical services looking to improve information sharing and service access for minors.

In Australia, the provision of gender care falls under the oversight and jurisdiction of individual states and territories^7^. Young people can therefore pursue a number of different clinical pathways to access gender care. Tertiary paediatric gender services are located in metropolitan areas across the country and provide access to care and support for young people and their families. Some young people may also decide to access community and/or mental health services depending on their location, ability to access services, and financial resources. Young people aged 17 and up may have their care managed by a general practitioner (GP) with specialist support (e.g. psychologist, endocrinologist). While these primary and secondary services are covered by Medicare (the Australian universal funding scheme), many services charge a fee which can be significantly greater than the Medicare coverage creating a “gap” fee that becomes an out-of-pocket expense for the patient^8^.

Gender care for minors (under 18) is also directed by case law at the national level. In 2020, the legal case *Re Imogen* established the requirement of consent from both legal parents for a young person to access gender care^9^. This decision raised the bar for parental consent from one parent to requiring two parents to provide consent in this specific area of healthcare^10,11^. While this ruling has now been superseded by more recent case law returning clinical consent to usual practice^12–14^, *Re Imogen* is the legal framework within which majority of our participants accessed gender care.

## Methods

### Study design

This paper, which forms part of a larger doctoral project, aims to answer the following research question: what do young people know about the process of accessing gender care? Critical phenomenology was used as the methodological framework for this project. Critical phenomenology remains grounded in lived experience without abstracting the historical and social structures that constitute such experience^15^. An understanding of young people’s lived experience seeking out gender care would be incomplete without critical attention to the larger systems that structure this experience. Similarly, systems do not simply exist or impede young people but are actively experienced and negotiated. Critical phenomenology therefore goes beyond describing lived experience to bridge experience with transformative thinking^15^.

Research design and methods were informed by indigenous tenets, such as equity in partnership, ownership, and self-determination^16,17^ as well as best practices for conducting research and working with transgender and gender diverse communities^18,19^. For example, an advisory group, which included young adults with lived experience of accessing gender care as minors, provided feedback on the project’s design, interview questions, and preliminary data analysis^16,18^. Similarly, participants were invited to choose their own pseudonym as a means to own their stories and to be able to find themselves within research publications^16,18^. Finally, attention to self-determination led to the use of open text fields for participants to self-identify in the demographic data form, and research participation that was guided by participants’ capacity to engage in sensitive research^17,20^.

## Participants

Participants were individuals in Australia aged 18–25, who had tried (successfully or not) to access gender care when they were under the age of 18. Individuals who were under the age of 18 at the time of recruitment were not eligible to participate. The eligibility criteria aimed to capture a diverse set of clinical experiences (e.g. young people who were unable to proceed with care, due to a lack of parental consent or system constraints, as well as those who were able to proceed) and to recruit participants who could reflect on their recent and complete experience as a minor. Recruitment took place through LGBTQ + and transgender-specific community organisations’ online channels (websites, social media, email, etc.), which posted notices on the researcher’s behalf, and by printed flyers in adult gender services. Individuals interested in participating were invited to contact the researcher through the project website. There was no direct person to person recruitment.

## Data collection

One-hour semi-structured interviews were conducted over Zoom by MS. The interview guide included questions which aimed to explore what participants knew or assumed about the process of accessing gender care when they were at the early stages of seeking care. Example follow-up questions included: if and how participants prepared for appointments, where participants looked for information, and who provided this information or supported them in this process. Interviews were audio recorded and transcribed verbatim. Interviews were de-identified using participants’ pseudonyms and all third-party information was removed at the point of transcription. Participants’ pseudonyms were held in strict confidence with the researcher.

Harm prevention was central to our research design and data collection process. Following discussions with the advisory council, participant emotional resiliency check-ins^20^ were conducted by a peer worker for each participant prior to their interview. Participants were able to choose to cancel or reschedule their interview at this point, but none took up this option. Steps were also taken throughout the interview to prevent harm (e.g. participants were offered breaks and reminded that they were free to skip questions), and a distress protocol was created. At the end of each interview, participants received a debrief package which included mental health resources and community support services which they could access without cost after their research participation.

### Data analysis

Data were coded and analysed using reflexive thematic analysis. Reflexive thematic analysis is a flexible and iterate process of interpretation and knowledge production^21,22^. Coding started with re-reading and familiarisation of the dataset. Following familiarisation, a first round of coding produced inductive, descriptive codes. MS conducted initial coding on all transcripts, then discussed the coding with two other members of the research team, working with a small number of transcripts for each iteration of this process. As a result of discussion, the coding scheme was clarified and expanded which led to a more active interpretation of the data, shifting the coding process along the analysis “continuum” to create latent codes^21,23^. In the next step, codes were grouped into theme by MS and again discussed with two other members of the research team. In this discussion, the themes and sub-themes were re-considered and revised before being finalised by MS.

## Ethics

As this research was conducted as part of a dual PhD program across two institutions, ethics approval was provided by both the University of Manchester (2023-16240-31171) and the University of Melbourne (2023-27280-47934-5) and conducted in line with university research guidance^24^, the Declaration of Helsinki^25^, and the Australian National Health and Medical Research Council’s National Statement on Ethical Conduct in Human Research^26^. Participants provided informed consent at two time points – written consent to join the research project and verbal consent at the start of their interview. Participants were free to withdraw from the project at any point.

## Results

### Participant characteristics

14 participants from four different Australian states participated in an interview (Table 1). Participants were aged 18–22 years old and accessed a variety of services. Participants were asked to complete a participant demographic form with open text fields to allow for self-identification. Gender identity is reported as shared with the research team alongside participants’ pseudonyms and quotes.

**Table 1:** Participant demographics.

Characteristic	Number of participants
*Age*	
18 19 20 21 22	4 4 2 3 1
*Gender Identity*	
Transgender male Trans man Queer-trans man Trans masculine Male Transgender woman Woman Female Nonbinary	2 4 1 2 1 1 1 1 1
*Ethnicity*	
Australian and Lao White/Persian White/Jewish Japanese Australian White/Caucasian Australian White/Caucasian Anglo-Celtic Greek heritage No response	1 1 1 1 3 4 1 1 1
*Disability/Neurodiversity*	
No	6
Yes	8
- Autism/ASD: 4 - ADHD: 3 - Dyslexia: 1 - Anxiety: 1 - Depression: 2 - OCD: 1	
*Current Occupation*	
Full time employee Part-time, Casual employee Freelancer Student (part/full time) Not currently working No response	1 6 2 10 1 0
*Geographic location*	
Urban Rural No response	13 0 1
*State/Territory*	
Western Australia Victoria New South Wales Queensland	3 4 3 4

## Themes

Three major themes were identified: (1) information seeking, (2) a lack of information affects expectations, and (3) a lack of information impacts decision making about accessing care.

1. *Information seeking*.

Participants described actively trying to find information about accessing gender care. For example, some participants did their own online research.

- So I found [a clinician] through Reddit and like community [sharing] doctor’s recommendations. – *Elizabeth (Transgender Woman*,* 18)*.
  I think I was doing some research about like hormones and stuff. Um I didn’t really have any friends who were currently at the [gender] service though, so I didn’t have the exact details… - *Helena (Female*,* 19)*.
  I also did a fair bit of research online as well like through different people’s sort of anecdotes of how they accessed various gender clinics. - *Jack (Trans Masculine*,* 19)*.

Some participants also relied on their parents or health professionals to help them find relevant information.

- Um in those early stages, I feel like my mum did a lot of the hard work [finding information]. A lot of networking with a lot of trans parents. – *Sam (Trans Man*,* 19)*.
  I had done plenty of my own research, of course. I think I had even been in contact with the gender service myself through email, just to be like, ‘hello [laughs] I didn’t know you guys exist, um, what’s kind of the deal?’- *Liam (Transgender Male*,* 21)*.

Interestingly, most participants described seeking information as a recurrent process – participants often sought more information about the services they wanted to access during waiting periods or had to find new information with each successive referral.

- during the 4 months [after attending a community health service] I did some research. I looked up this website that has a list of GPs [who specialise in gender care]… So I didn’t go to my local GP. I went to a different one. – *Jason (Trans Man*,* 21)*.
  I wasn’t really sure what the actual process involved other than go talk to this specialist. - *Icarus (Queer-Trans Man*,* 22)*.

While participants found that gathering information through these various channels provided some insight, they often encountered information that was lacking in specificity.

- Look obviously I watched plenty of YouTube videos [about other people’s experiences accessing care], but they were all from people from the UK or the US, so all very different systems. - *Liam (Transgender Male*,* 21)*.
  So, information wise, concerning what happens [to your body] when you get the care, I think I was good. But what it takes to get the care, I think that was where there was the information gap. - *Rocky (Nonbinary*,* 20)*.
  You can do as much research as you want online, and you will not actually find anything about actually how long waitlists are or like how many appointments, the actual requirements, everything… it was like oh, I just have to like jump in the deep end and find out sort of thing. – *Nathan (Trans Masculine*,* 18)*.

Despite participants’ efforts to find information about accessing gender care, most participants struggled to find relevant, locally specific information about the process of accessing care.

(2) *A lack of information affects expectations*.

Our participants did not know, overall, what to expect from clinical services.

- So I was pretty nervous cause I had like no idea what they were going to do or what they were going to ask me. Or anything. So I was just sort of going in completely blind.- *Nathan (Trans Masculine*,* 18)*.

Importantly, a lack of information about clinical services played an important role in participants’ expectation setting. On one hand, a lack of information was interpreted negatively by some participants. Participants were critical of why a gap in service information existed in the first place.

- [it] would have been really good to sort of have a more solid idea [of what accessing care looked like] rather than like that very smoke and mirrors aspect of it, where it’s very secret squirrels… I don’t think healthcare should be this secret club, only if you know, you know and if you don’t know, you don’t know. - *Jack (Trans Masculine*,* 19)*.
  I really didn’t have information about this process. There isn’t enough information about people who actually access this care. And the people who have actually accessed this care they really just give information on the downlow. So it was really, really a lot of trial and error.- *Rocky (Nonbinary*,* 20)*.
  I feel like [due to the waiting times], if you’re [young enough] for puberty blockers and not HRT, that is your only hope of getting gender affirming medical care under 18… which is a bit absurd because a lot of trans people only really start transitioning and coming to the realisation in their early to mid-teens. So, it feels like the system is very much against you. - *Sam (Trans Man*,* 19)*.

For these participants, a lack of clarity around clinical processes and wait times left them feeling that aspects of their care were kept secret or that the system was against them. On the other hand, the information that participants did find and engage with also played an important role in expectation setting, particularly around how participants considered interacting with their clinician.

- I feel like [due to the waiting times], if you’re [young enough] for puberty blockers and not HRT, that is your only hope of getting gender affirming medical care under 18… which is a bit absurd because a lot of trans people only really start transitioning and coming to the realisation in their early to mid-teens. So, it feels like the system is very much against you. - *Sam (Trans Man*,* 19)*.
  I guess during that time when I was doing the diagnosis, there was a big thing on rapid onset gender dysphoria. So, I was quite eager to dispel that this doesn’t apply to me. Um even though it’s something that the science behind it isn’t exactly rigorous. So that was my main concern at the time of I am going to see these experts in this field, and they are going to look at me and they are going to put this label on me. – *Cameron (Male*,* 19)*.
  I had seen people [online] talk about what they went through to get to that process, so I kind of had in my head, ‘okay they probably want me to say things like this.’ But, that lined up with what I would have said anyway. - *Liam (Transgender Male*,* 21)*.
  at the start, you talk about it in ways that you think that [clinicians] want to hear, especially back then there was a lot of rhetoric at the time of like the right way to be trans or whatever… I was very new to coming out and I had heard that rhetoric especially online. – *Cal (Trans Man*,* 20)*.

Together, these quotes illustrate that the information or sources that participants encountered while seeking out gender care could lead to feelings of uneasiness and apprehension towards clinicians.

(3) *A lack of information impacts decision making around accessing care*.

Participants explained that having more information about the process of accessing gender care would have allowed them to consider what would work best for them and their individual circumstance. For a couple of participants who had unsupportive parents, their inability to find information about clinical processes and the requirement for parental consent played a key role in their decision to pursue care as a minor.

- So, I should have just waited [until I was an adult] … And I think the process of when you want gender affirming care and your parents don’t agree with it. It gets really, really messy. Well in my case it was. - *Rocky (Nonbinary*,* 20)*.

In this way, limited information around requirements such as parental consent hindered participants’ ability to decide *if and when* they would like to engage their families, particularly where families may not be supportive and current wait times may limit access to paediatric services. A lack of information also impacted participants’ ability to plan and make specific decisions about their care.

- just everything about that process [of getting a psychologist letter] was like, what do I ask the psychologist? How do I work this out? Oh, I need to put some money aside for this as well when I was already saving to pay for the other appointments. – *Elizabeth (Transgender Woman*,* 18)*.
- I didn’t really know what was going to happen. I didn’t know how the gender clinic functioned really. I would have done things differently in hindsight if I knew how it actually functions. – *Lucy (Woman*,* 18)*.
- I mean it was um somewhat anxiety provoking going into it without any knowledge…You know I think its knowledge about what the process wants from you, so that you can find someone that best suits your needs because there are a lot of people with different approaches. - *Cameron (Male*,* 19)*.

Participants wanted readily available information about clinical processes in order to understand and prepare for potential costs and wait times, as well as consider and decide between different service options or clinicians’ approaches to care. A few participants gave clear suggestions regarding how clinical services could provide such information.

- Just more information about wait times. Look as far as I know you can’t just google what the wait times are, like obviously. And I understand why that is the case, but I think making it easier to understand what the wait times will be and whether that’s the best option for young people. - *Liam (Transgender Male*,* 21)*.
- Maybe a website, where if you want to access this care, you can actually get to talk to someone and ask the questions before you decide yes, I want to go along with this process. - *Rocky (Nonbinary*,* 20)*.
- I think maybe making trans people more visible within the community would be really helpful. So someone can say, ‘oh yes that person looks like me and has experiences like me. They’ve had this positive experience at a gender clinic. Maybe that means, I can go and get support from there too’…” – *Jack (Trans Masculine*,* 19)*.

Participants identified wanting more information particularly from the local services they were trying to access in order to make informed decisions around accessing care.

## Discussion

Previous research has identified that young people struggle to find information when accessing gender care^2–4^. Our findings provide further insight into this experience and increase clarity around the types of information young people want prior to accessing gender care.

We propose that there are at least three types of information that young people look for when trying to access gender care: (1) *medical* - information about medical interventions (physical changes, side effects, etc.), (2) *experiential* – information from peers about what the process or experience will be or feel like, and (3) *service* – information about how clinical services work (wait times, number of appointments, requirements, etc.). A similar typology has been described by Jaaniste, Hayes, and Von Baeyer for information provided to paediatric patients prior to medical procedures^27^. In our cohort, these differing types of information were primarily sought through the internet and social media which reflects other research findings on the ways young people look for and engage with health information^2,28–30^. Participants engaged with medical information and experiential information shared by peers to understand and feel better prepared to access gender care. While medical and experiential information provided some (albeit limited) insight, our participants primarily discussed a lack of information about the services they were trying to access. These findings suggest that the lack of information as a barrier to accessing gender care that has previously been identified in the literature primarily stems from a lack of *service* information. Participants described the significant role that a lack of information about the clinical services they were trying to access played in the uncertainty they felt and the challenges they faced when trying to access gender care as a minor.

Our findings also point to the value of providing locally specific service information to young people. Participants often described trying to piece together or make sense of service and experiential information far beyond the Australian context. While a lack of Australian-specific experiential information may highlight participants’ initial lack of connectedness to community channels or networks at these early stages of seeking out information^31^, a gap in service information is particularly concerning as the landscape around gender care continues to evolve. It is important for clinicians working in this area to consider how personal online anecdotes and changes to care provision internationally may ‘fill the gap’ when accurate, locally specific service information is not readily available. In fact, some participants relied on media and online rhetoric they encountered prior to accessing care (e.g. rapid onset gender dysphoria and the “right way to be trans”) to try to anticipate their clinicians’ knowledge and understanding of transgender and gender diverse experience. For young people facing increasing hostility before they even enter the clinic, sharing locally specific service information may help young people find accurate and reliable information at an early stage^29,32^. Additionally, sharing clinic-specific experiential information (e.g. in the form of patient testimonials on service websites) may allow clinical services to provide young people with accurate information in a way that is relevant and meaningful to them^30–32^. Engaging with young people in the development of relevant service information resources will be a vital step towards improving service transparency^16,19^.

While there are practical reasons for providing accurate service information at an early stage, there are also strong ethical reasons for clinicians and clinical services to consider sharing such information. Firstly, greater transparency around clinical processes respects and supports young people’s developing agency^33^. Participants described wanting service information to be able to understand what to expect and make decisions about their care. Information that allows young people to be able to manage their own expectations, prepare for upcoming costs, and understand timelines provides valuable opportunities for young people to be active partners in their own care^34,35^. Similarly, providing information prior to service access creates an opportunity for young people to make informed decisions around what will be the safest or most appropriate approach from them in seeking care, such as engaging their parents at an early stage or perhaps waiting to access adult services.

Secondly, providing young people with accurate, age-appropriate information may establish a strong foundation for the therapeutic relationship. In our findings, a lack of clinical transparency was interpreted as secrecy or the system being against patients, which seemed to undermine trust before young people even encountered a clinician. These findings reflect other research that shows that a lack of information, particularly when wait times are long, contributes to feelings of uncertainty and decreases trust in clinical staff and the wider healthcare system^36,37^. In a similarly contentious area of healthcare such as abortion care, a lack of reliable, readily available information has been shown to impact the therapeutic relationship, patient wellbeing, and expectations^38,39^. Services that provide relevant and reliable information about their clinical processes prior to service access may address these dynamics early on and help engender trust and transparency between young people and their clinicians.

It is also important to consider the challenges faced by clinicians and clinical services around information provision prior to service access. In paediatric or adolescent gender care where service information has previously been misrepresented^40^, clinical services may be hesitant to make information about their clinical processes widely available. In addition, limited funding and resources impact service capacity and wait times^41^. Providing accurate and up-to-date service information may quickly become a low priority for clinicians when resources are already constrained. Similarly, clinical services and clinicians might see the request for information prior to service access beyond the bounds of the therapeutic relationship (i.e. before a young person becomes a patient). While these concerns require careful thought and consideration, our findings here suggest that clinical services should seriously consider participants’ desire for service information at an early stage and the impact a lack of such information can have on patients and the therapeutic relationship. Moreover, while medical and experiential information is currently and arguably appropriately disseminated through various health professional associations, community websites, and social media platforms, the ability for young people to access accurate, reliable, and locally specific service information hinges on the services themselves making it available. Clinicians and clinical services are therefore best placed to meet this need and provide service information to young people.

### Limitations

Despite recruitment efforts to promote diversity, the majority of participants identified as white and male or transgender men. In addition, all participants lived in urban locations and are therefore likely to have had very different experiences accessing health services compared to their rural counterparts^42^. Further research is needed to reach these populations.

## Conclusion

This paper sheds further light on the information young people seek out and encounter prior to accessing gender care as a minor. Young people are actively engaged in the process of accessing gender care and want greater transparency from the services they are trying to access. Clinical services are best placed to provide accurate, age-appropriate, and locally specific service information to young people prior to accessing gender care. From a practical and ethical point of view, providing such information may facilitate a young person’s decision making and support the therapeutic relationship at an early stage. Further research should look at the clinical feasibility and implementation of sharing service information, as well as the impact such information has on young people trying to access gender care.

## Data Availability

The datasets generated and/or analysed during the current study are not publicly available due to reasons of sensitivity but are available from the corresponding author on reasonable request. All requests will be subject to research ethics committee approval, as per the ethics approval conditions for the study.
